# New Discovery of Natural Zeolite-Rich Tuff on the Northern Margin of the Los Frailes Caldera: A Study to Determine Its Performance as a Supplementary Cementitious Material

**DOI:** 10.3390/ma17174430

**Published:** 2024-09-09

**Authors:** Jorge L. Costafreda, Domingo A. Martín, Miguel A. Sanjuán, Jorge L. Costafreda-Velázquez

**Affiliations:** 1 Escuela Técnica Superior de Ingenieros de Minas y Energía, Universidad Politécnica de Madrid, C/Ríos Rosas, 21, 28003 Madrid, Spain; domingoalfonso.martin@upm.es; 2 Laboratorio Oficial Para Ensayos de Materiales de Construcción (LOEMCO), C/Eric Kandell, 1, 28906 Getafe, Spain; 3 Department of Science and Technology of Building Materials, Civil Engineering School, Technical University of Madrid, 28040 Madrid, Spain; masanjuan@ieca.es; 4 Department of Constructions, University of Holguín, Avenida XX Aniversario, Vía Guardalavaca, Piedra Blanca, Holguín 80100, Cuba; costafreda1992@gmail.com

**Keywords:** mordenite, smectite, SiO_2_ reactive, CaO reactive, cement, mortars, pozzolanicity, mechanical strength

## Abstract

The release of Neogene volcanism in the southeastern part of the Iberian Peninsula produced a series of volcanic structures in the form of stratovolcanoes and calderas; however, other materials also accumulated such as large amounts of pyroclastic materials such as cinerites, ashes, and lapilli, which were later altered to form deposits of zeolites and bentonites. This work has focused on an area located on the northern flank of the San José-Los Escullos zeolite deposit, the only one of its kind with industrial capacity in Spain. The main objective of this research is to characterize the zeolite (SZ) of this new area from the mineral, chemical, and technical points of view and establish its possible use as a natural pozzolan. In the first stage, a study of the mineralogical and chemical composition of the selected samples was carried out using X-ray diffraction (XRD), scanning electron microscopy (SEM), X-ray fluorescence (XRF), and thermogravimetric analysis (TGA); in the second stage, chemical-qualitative and pozzolanicity technical tests were carried out at 8 and 15 days. In addition, a chemical analysis was performed using XRF on the specimens of mortars made with a standardized mixture of Portland cement (PC: 75%) and natural zeolite (SZ: 25%) at the ages of 7, 28, and 90 days. The results of the mineralogical analyses indicated that the samples are made up mainly of mordenite and subordinately by smectite, plagioclase, quartz, halloysite, illite, and muscovite. Qualitative chemical assays indicated a high percentage of reactive silica and reactive CaO and also negligible contents of insoluble residues. The results of the pozzolanicity test indicate that all the samples analyzed behave like natural pozzolans of good quality, increasing their pozzolanic reactivity from 8 to 15 days of testing. Chemical analyses of PC/SZ composite mortar specimens showed how a significant part of SiO_2_ and Al_2_O_3_ are released by zeolite while it absorbs a large part of the SO_3_ contained in the cement. The results presented in this research could be of great practical and scientific importance as they indicate the continuation of zeolitic mineralization beyond the limits of the San José-Los Escullos deposit, which would result in an increase in geological reserves and the extension of the useful life of the deposit, which is of vital importance to the local mining industry.

## 1. Introduction

There are many studies in Spain where natural zeolites are mentioned in relation to genetic and spatial bentonite formations of volcanic origin [1,2,3,4,5]. Authors such as Mattei et al. [6], De la Fuente and Linares [7], and López-Ruiz and Cebriá [8] explain in detail the formation processes of bentonites and highlight the affinity between clay minerals, such as smectites and mordenite, assuring that their origin is typically hydrothermal and related to the volcanism of southeastern Spain. Other researchers [9,10] mention the association of zeolite with bentonite formations located in known deposits in southeastern Spain, such as Morrón de Mateo, Los Trancos, and the proximal surroundings of San José and Los Escullos. De La Villa et al. [11] state that these zeolites may have been formed during the alkaline reaction processes of bentonite. Deon et al. [12] detected zeolite associated with layers of illite and smectite in their investigations in the Rodalquilar caldera in the southeast of the Iberian Peninsula. In the report of the Geological Map of Spain [13], zeolite is referred to as a residual material of the bentonitization process, along with other minerals such as smectite (montmorillonite variety), quartz, plagioclase, calcite, amphibole, mica, and tridymite. Despite the above, the most detailed and specific work on the study of zeolite began in 2005 [14,15,16], with important contributions not only in mineralogical characterization but also in heat treatment to improve its technological properties, which was also stated by Suárez et al. [17]. However, despite the fact that zeolitic mineralization is practically ubiquitous in the volcanic environment of the Neogene, some authors have confirmed that the highest concentration of zeolite of the mordenite variety occurs in Los Frailes caldera [18], where the only industrial zeolite deposit in Spain is found. In fact, Martín et al. [19] have proven by electrical resistivity tomography (ERT) that the thickness of the mineralized horizon of this deposit exceeds 40 m. The most recent applications of zeolite from this region have been aimed at the chemical stabilization of wastewater from mining and metallurgical processes where there is an abundant presence of heavy metals [20]. Uses in the manufacture of waste-based compost for soil improvement are also reported due to its effectiveness in retaining metals and ammonium [21]. Domene et al. [22] have effectively restored vegetation in semi-desert areas of Almeria (Spain) through the standardized use of organic compost prepared with a mixture of zeolite and greenhouse waste from that region. Possibly the most widespread application of this zeolite in recent times is as a highly reactive pozzolan for the improvement of cements, mortars, and concretes [23,24]. This is due to the need to control and reduce CO_2_ emissions into the atmosphere. In this sense, Byung-Wan et al. [25] designed zeolite mortars using an alkaline activator (NaOH), obtaining a mechanical strength of 43.5 MPa at 7 days. This is essentially due to the mineralogical composition, texture, structure, physical, and mechanical properties of the zeolites [26]. Iswarya and Beulah M [27] carried out an extensive review on the specific use of zeolite in the manufacture of high-strength concretes. The use of zeolite in the manufacture of high-strength concrete is a common practice today, resulting in lighter structures that are resistant to sulphate attack, seawater, and frost [28,29,30]. Finally, Boháč et al. [31] and Vejmelková et al. [32] have shown that in zeolite-containing cementitious pastes, an acceleration of hydration occurs much faster than the time at which the peak of the exothermic flow occurs.

**Figure 1:**
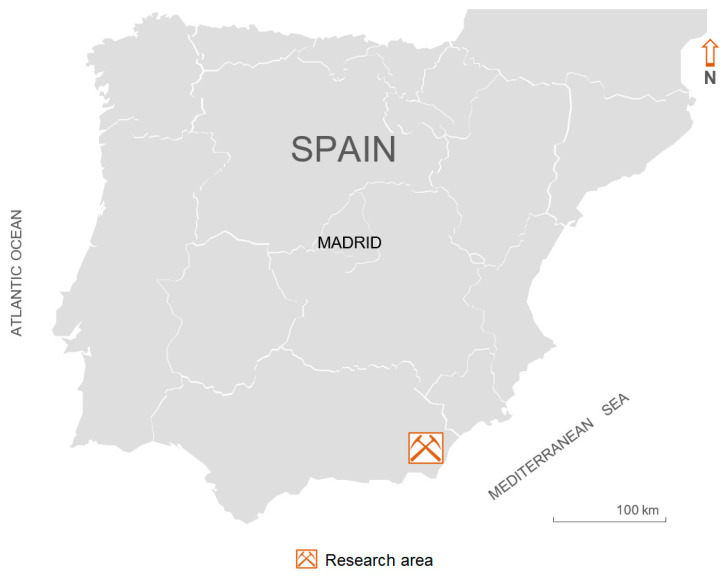
Location of the research area.

The main objective of this work is the mineral, chemical, and technical characterization of the natural zeolite-rich tuff from new evidence discovered in an unexplored area within the Los Frailes caldera and to prove that it is sufficiently pozzolanic in capacity to be used effectively as a reactive aggregate in mortars and concretes. The second objective is to demonstrate that these materials have properties similar to those of the main deposit known as San José-Los Escullos, located 655 m to the south (Figure 1), which would have a positive impact on the expansion of the mining area and an increase in geological reserves.

## 2. Materials and Methods

### 2.1. Materials

In this research, 6 natural zeolite-rich tuff samples weighing 20 kg each were used. The specimens are made of a zeolite tuff of pale green, light gray, and white. It is usually compact, although in parts it is friable and has a low density. It forms extensive outcrops embedded in andesitic and dacitic rocks. All the samples were carefully crushed and sifted to the particle size fraction of 63 μm. In addition, a Type I Portland cement was used, the chemical composition of which is shown in Table 1.

In the dosage of the mortar, normalized sand (NS) was used; this sand is made of quartz of rounded grains, where the SiO_2_ content is 98%. The granulometric distribution of normalized sand (NS) is listed in Table 2.

Table 3 shows details of the proportions of materials used in both the mixed mortar specimens (PC/SZ) and the reference specimen (PCSR).

### 2.2. Methods

A study of the mineralogical phases in the samples was carried out by X-ray diffraction (XRD) with the help of the Rigaku Miniflex-600 diffractometer of the Escuela Técnica Superior de Ingenieros de Minas y Energía (Universidad Politécnica de Madrid, Madrid, Spain). The study of the phases was performed in an interval of 4° to 60°, with a step of 0.01° and at every 5° intervals. A voltage of 40 kV and a current of 15 mA were applied.

A scanning electron microscopy (SEM) study was carried out to determine the morphological properties of the minerals in the samples, such as the crystalline texture, species, and size as well as surface characteristics and the presence of pores, cavities, and channels. A Hitachi S–570 electron microscope was used from the Centralized Laboratory of the Escuela Técnica Superior de Ingenieros de Minas y Energía (Universidad Politécnica de Madrid, Madrid, Spain). The microscope is equipped with a Kevex-1728 analyzer, a BIORAD Polaron, a power supply for evaporation, and a Polaron SEM coating system. It has a resolution of 3.5 nm and an amplification of 200 × 10^3^.

The chemical composition of the samples in their natural state was determined by X-ray fluorescence (XRF). Compounds such as SiO_2_, Al_2_O_3_, CaO, Na_2_O, K_2_O, MgO, Fe_2_O_3_, TiO_2_, and MnO were found. In addition, loss on ignition (LOI), Si/Al ratio, and Si/(Al + Fe) ratio were determined. This test was also carried out on mixed mortar specimens (cement/natural zeolite-rich tuff) hardened at different ages (7, 28 and 90 days) to determine the behavior of compounds over time. A Philips WDXRF spectrometer (PW1404) was used. The intensity of the radiation ranged from 10 to 100 kV. This equipment belongs to the Universidad Politécnica de Madrid (Madrid, Spain).

Thermogravimetric analysis was carried out to study the thermal behavior of the samples and establish the main thermal events that take place until their definitive collapse. The samples were analyzed by (TGA) with air atmosphere and at 20 °C/min. The heating range was 25 °C to 950 °C. This analysis was carried out in the Laboratorio de Tamices Moleculares of the Instituto de Catálisis y Petroleoquímica of the Centro de Investigaciones Científicas (CSIC) of Madrid (Madrid, Spain).

The pozzolan quality of the zeolite samples was determined by a qualitative-technological chemical analysis, following the specifications of the Standard UNE–EN 196-2-2014 [33]. The main objective of this test is to determine the percentages of reactive SiO_2_ in relation to the total SiO_2_ present in the sample capable of reacting with Portland cement in the hydraulic reaction process. Other compounds such as total-reactive CaO, MgO, Al_2_O_3_, Fe_2_O_3_, and SO_3_ were determined. The proportion of insoluble residue (IR) and loss on ignition (LOI) of each sample were calculated. This study was carried out at the Laboratorio Oficial de Ensayos de Materiales de Construcción (LOEMCO), Getafe (Spain).

The pozzolanic reactivity of the samples was calculated by chemical analysis of pozzolanicity. This test takes into account the reaction capacity of zeolite with the Ca(OH)_2_ present in a solution together with a certain amount of Portland cement. In total, 75% Portland cement and 25% zeolite were mixed. The assay considers the variation in the concentration of hydroxyl ions and calcium ions in solution over a period of 8 and 15 days [34].

A mechanical compressive strength test was carried out on specimens made with a mixed mixture of Portland cement and natural zeolite-rich tuff. A type 1 Portland cement, class 52.5 R, was chosen, whose characteristics are detailed in the Standard UNE–EN 197-1:2011 [35]. Standardized sand was used as a fine aggregate to produce the mixed mortar specimens hardened to 7, 28, and 90 days in a cement to natural zeolite-rich tuff ratio of 75:25% [36]. Distilled water was used in the dosing of the paste. The proportion of materials in the cement/natural zeolite-rich tuff mortars was as follows: cement 375 g, zeolite: 125 g, fine aggregate (standard sand Type CEN EN 196-1 [36]): 1350 g, and distilled water: 225 g. In the reference mortars (Portland cement only), the formulation was PC: 450 g, fine aggregate: 1350 g, and distilled water: 225 g. The mortar specimens were placed in a container with water in a humid chamber at a temperature of 20 °C ± 1 °C.

## 3. Results and Discussion

### 3.1. X-ray Diffraction (XRD)

**Figure 2:**
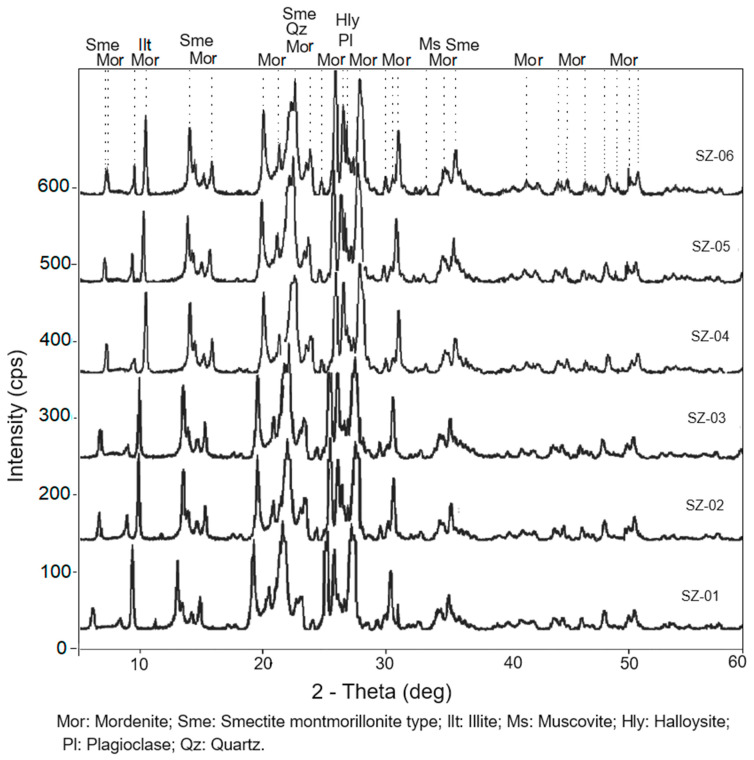
X-ray diffraction patterns of the samples of natural zeolite-rich tuff.

The results of XRD analyses revealed a major presence of a crystalline phase consisting of mordenite. The secondary phases were smectite (montmorillonite), illite, halloysite, quartz, plagioclase, and muscovite (Figure 2).

The morphology and distribution of the various peaks of mordenite indicate a highly developed degree of crystallinity in relation to the other phases.

The mentioned mineral phases have also been found in the main deposit (San José-Los Escullos) by other researchers [18], who have established that the content of mordenite in the natural zeolite-rich tuff formations of the deposit ranges from 47 to 95%. However, they describe a wider variety of mineralogical phases not found in the study area, such as orthoclase, sanidine, calcite, hematite, illite, chlorite, and pyrite. These same phases have been located at 40 m depth in the Los Frailes caldera through the study of drill cores and geophysical work by Martín et al. [19]. On the other hand, Stamatakis et al. [1] have found opal and cristobalite, which they attribute to the action of residual fluids enriched in silica.

The newly discovered area could be considered an extension of the zeolitic mineralization towards the northern flank of the deposit.

### 3.2. Scanning Electron Microscopy

**Figure 3:**
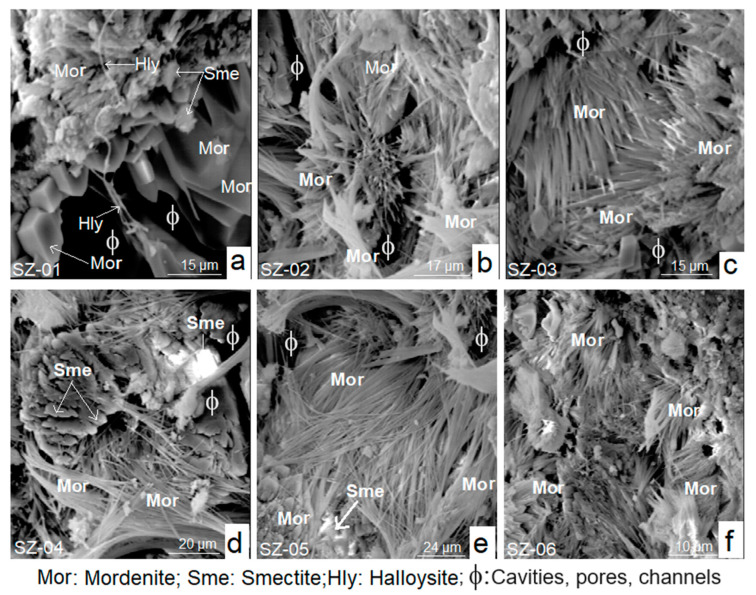
SEM micrographs (**a**–**f**) of the analyzed samples.

The results obtained by SEM showed a major presence of mordenite crystals in relation to smectite and halloysite (Figure 3a–f). From a morphological and textural point of view, mordenite exhibits markedly idiomorphic textures. Its habits are preferably acicular, fibrous, rhombohedral-hexagonal, and sometimes bacillary. The crystals crisscross each other to form very compact radial aggregates. The spatial arrangement of aggregates often forms cavities and intergranular voids.

The crystals of mordenite are frequently intergrown with smectite; both species are formed from the alteration of amorphous materials [18,19]. According to Stamatakis et al. [1] the mordenite that appears in the main deposit in the form of fibrous crystals has formed at the expense of glassy matter of volcanic origin.

### 3.3. X-ray Fluorescence (XRF)

The data obtained from the XRF analysis showed high SiO_2_ and Al_2_O_3_ contents (Table 4). In addition, the contents of the alkaline compounds (Na_2_O and K_2_O) were significantly higher than those of the alkaline–earth compounds (CaO and MgO). The contents of SO_3_ were practically negligible. The calculation of the Si/Al ratio showed values in the range of 4.0 to 5.2, which indicates that the samples studied have pozzolanic properties [37,38]. The Si/(Al + Fe) ratio ranged from 3.6 to 3.9. Loss on ignition varied from 10.7% to 11.9%. The ratio between SiO_2_ and the alkaline and alkaline–earth compounds was high.

It is inferred that the low CaO and MgO contents in the samples are due to the fact that Ca^2+^ and Mg^2+^ cations are removed during hydrothermal processes [39], which confirms the hydrothermal genesis of zeolite deposits in southeastern Spain [40]. However, for the same reason, the contents of SiO_2_, Na_2_O, and K_2_O tend to increase [18].

### 3.4. Thermogravimetric Analysis

**Figure 4:**
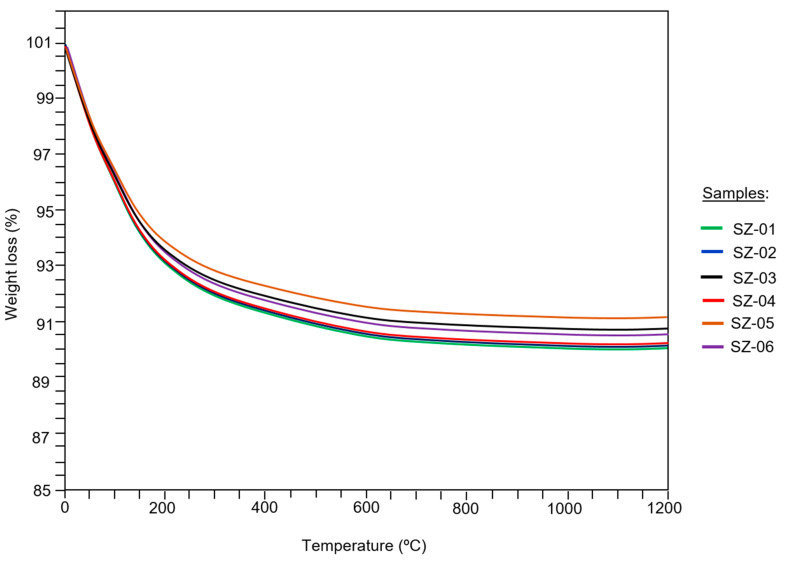
Behavior of the TGA curves in the samples studied.

The behavior of the TGA curves showed a simple decomposition process of the samples, with three well-defined thermogravimetric events (Figure 4). The first event occurred from relatively low temperatures (0–39 °C) to 250 °C, with a mass loss equivalent to 8.5% and 7.6%. In this interval, all the samples experienced rapid moisture loss and surface dehydration with the expulsion of the gases contained in the pores. The second event was recorded in the thermal range of 250 °C to 750 °C, with a mass loss of 2.3% to 1.2%. In this interval, the process of dehydration of the samples continued, possibly due to the loss of intrareticular or zeolitic water from mordenite and smectite; however, there were still no signs of structural collapse. In this thermal range, the dehydroxylation of the smectites occurred by the disintegration of the group (OH)^−^. The third and last thermal event occurred between 750 °C and 1200 °C, the fundamental feature being the stabilization of the TG curve. At this stage, the structural rearrangement of the samples occurred without processes of mass loss or gain.

Research carried out at the San José-Los Escullos deposit established that the zeolite from that deposit behaves similarly to those in this work when subjected to thermal analysis, the most notable feature being the simple decomposition in three consecutive stages [41]. However, the percentage of mass loss of zeolite from the main deposit in the first thermal event was much lower, which can be interpreted as the zeolites being of a higher purity in this research.

### 3.5. Chemical Analysis of Technical Quality

The results obtained through the chemical-technological test showed that the samples behave like natural pozzolans capable of reacting hydraulically with Portland cement (Table 5). As far as could be seen, virtually all the SiO_2_ was reactive in all the cases. For example, in sample SZ-01, the total SiO_2_ calculated was 69.25%, of which 68.22% was the part that reacted to the cement; that is, more than 98% of the original SiO_2_ in this sample was reactive. This was also proven in the remaining samples. A similar fact has been verified with reactive CaO in relation to total CaO. According to established standards, a sample is chemically suitable from a qualitative-technological point of view if its SiO_2_ content > 25%, Al_2_O_3_ < 16%, MgO < 5%, SO_3_ < 4%, and insoluble residue (I.R.) < 3% [35]. The values calculated and shown in Table 5 reflect this approach.

Presa et al. [41] have calculated reactive SiO_2_ contents for zeolite from the San José-Los Escullos deposit in the range of 58.68% to 63.16%, which are comparatively lower than those of the samples analyzed in this work. These authors also calculated between 14.08% and 19.87% of insoluble residue present in their samples, percentages that are significantly higher than those calculated in this work. These arguments seem to confirm that the zeolite found in the new study area is much purer than that lying in the main deposit.

### 3.6. Chemical Analysis of Pozzolanicity

**Figure 5:**
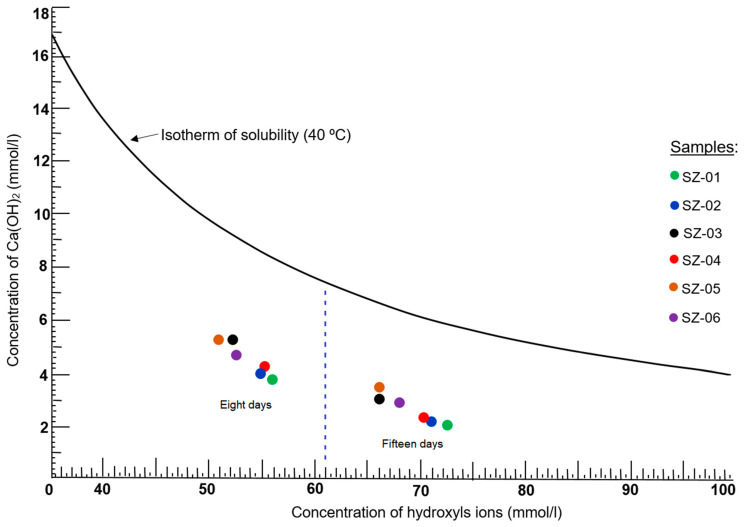
Evolution of the pozzolanic behavior of each sample analyzed at 8 and 15 days.

Figure 5 shows that all the samples occupied a deep position in the area below the isotherm of CaO solubility at 40 °C. This fact emphasises that all the samples analyzed were pozzolanic [34]. According to this, it appears that samples SZ–01, SZ–02, and SZ–04 were the most pozzolanic. If this fact is compared with the data shown in Table 5, the highest SiO_2_ contents corresponded precisely to these samples.

**Figure 6:**
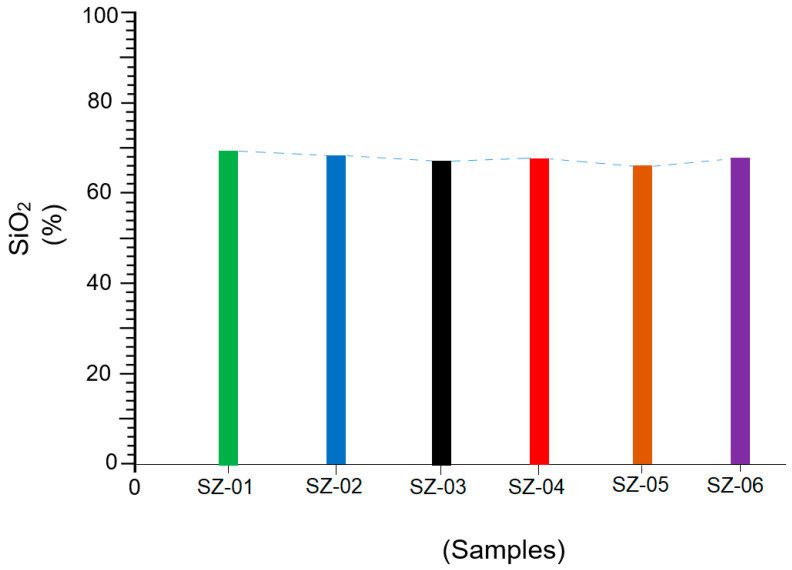
Behavior of SiO_2_ in the analyzed samples.

It could then be established that the SiO_2_ content is a decisive factor in the pozzolanic behavior of a material [42] (Table 5 and Figure 6). Authors such as Caputo et al. [43], Saraya and Thabet [44], and Mertens et al. [45] also state that more siliceous zeolites are more pozzolanic and contribute to the gain in mechanical strength. However, other factors must be considered, such as the amount of reactive CaO present, Al_2_O_3_, and the relationship between the compounds SiO_2_, CaO, and MgO [46]. It should be noted that the pozzolanic reactivity of the samples experienced notable increases from 8 to 15 days, according to the graph in Figure 5.

Figure 6 shows the variation in the SiO_2_ content for each sample analyzed. Note that those samples with higher silica contents are the same ones that in Figure 5 have higher pozzolanic reactivity, both at 8 and 15 days.

### 3.7. Chemical Analysis of Mortar Specimens

In this research, mortar specimens hardened at 7, 28, and 90 days were studied to monitor and compare the behavior of chemical compounds in both types of specimens over time. These specimens were made from a mixture of 75% Portland cement and 25% natural zeolite-rich tuff. In this case, the SZ-01 sample was used as a representative. In addition, reference mortars with exclusive Portland cement content were manufactured.

Table 6 shows the behavior of the different chemical compounds of anhydrous Portland cement and their mixtures in mortars set at 7, 28, and 90 days. The first detail to highlight is the variation in the SiO_2_ content (47.31%) of the Portland cement specimen at 7 days of setting in relation to the initial composition (17.47%). This increase was due to the presence of normalized sand [47], which contributed silica to the paste; however, a gradual decrease was observed at 28 and 90 days, respectively. A similar but less notable case occurred with Al_2_O_3_. The CaO experienced a noticeable decrease after 7 days of setting, practically half of the original content. The other compounds, with the exception of MgO, which experienced a small increase, tended to decrease between 7 and 90 days of setting. As shown in Table 6, this is the normal behavior of a Portland cement mortar without pozzolanic additions [18,36].

Table 7 shows the behavior of the chemical compounds of Portland cement and natural zeolite-rich tuff when both were mixed in a 75:25% ratio. After 7 days of setting, a decrease in SiO_2_ was observed in the specimens. This decrease still occurred at 28 and 90 days. Al_2_O_3_ also decreased in relation to its original values; its presence could prevent the C_3_A of the cement from reacting completely, thus preventing the progressive formation of ettringite, which would favor the hydration of bicalcium and tricalcium silicates [48]. 

If the original CaO contents are compared with those observed at 7, 28, and 90 days, a notable increase is observed (Table 7). It seems then that in the hydraulic reaction process, 48.63% of SiO_2_ was consumed after 7 days, 47.30% at 28 days, and 42.71% at 90 days. The sum of these percentages far exceeds 65.65% of the natural zeolite-rich tuff sample, so it follows that the system takes SiO_2_ from both zeolite and normalized sand. It is evident that this excess of SiO_2_ in the paste would imply a greater hydraulic reaction over time with the associated increase in the mechanical strengths of the mortar specimens. Previous studies at the main deposit have shown that PC/zeolite composite mortar specimens achieve mechanical strengths in excess of 51 and 72 MPa at 28 and 90 days, respectively [18]. 

The SiO_2_/CaO ratio is then inversely proportional, from which it follows that to fix more lime, it is necessary to consume more silica, giving tobermorite as a reaction product [18,48]. The Na_2_O/K_2_O ratio in the samples was lower than that in the reference cement.

It follows that more silica was available at the cement to natural zeolite-rich tuff interface than at the interface where there was only cement. This availability of SiO_2_ could favor an increase in mechanical strengths in the long term, as discussed above. Some researchers mention that the presence of pozzolans of zeolitic origin leads to an increase in the compressive strength of mortars and concretes after 28 days of setting, even exceeding the resistance of ordinary cement at older ages [49]. The SO_3_ content in specimens made with natural cement to natural zeolite-rich tuff mixtures decreased significantly over time. As can be seen in Table 7, the amount of SO_3_ from 7 to 90 days was comparatively lower than that recorded in specimens made of cement alone (Table 6). In the research of some authors, this fact is confirmed in a wide variety of natural pozzolans [50,51,52].

### 3.8. Mechanical Compressive Strength Tests at 2, 7 and 28 Days

**Figure 7:**
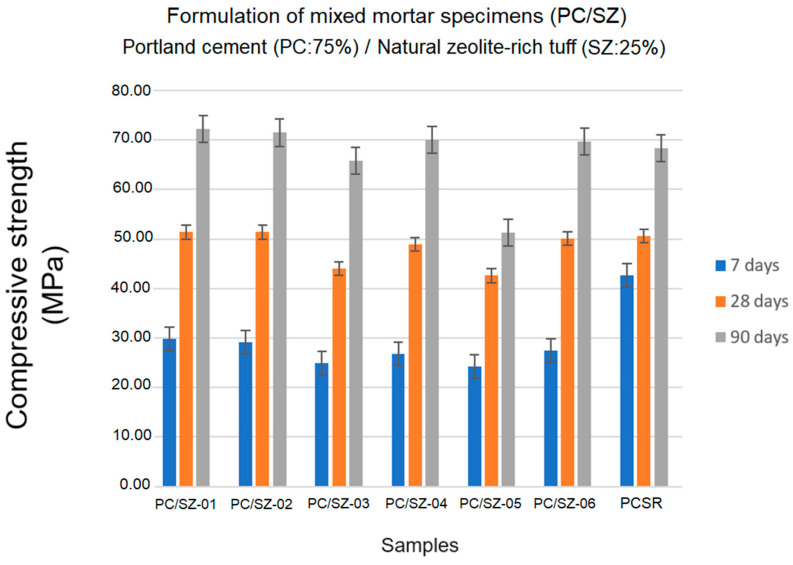
Behavior of the mechanical resistance in the period of 7, 28, and 90 days of curing.

Figure 7 shows the results of the mechanical compressive strengths calculated at 7, 28, and 90 days. An increasing rise in strength was observed for all the specimens made with a mixture of Portland cement and natural zeolite-rich tuff. Accordingly, it is once again confirmed that the presence of zeolite as a pozzolan favors the hydraulic reaction in the paste [45,53,54,55]. Furthermore, the capacity of zeolite to substitute Portland cement (PC/SZ: 75–25%) effectively without negatively altering the rheological properties of the mortars was tested.

As shown in Figure 7, after 7 days of curing, all the specimens had lower mechanical strengths than the standard specimen (PCSR: 42.7 MPa). However, their values were relatively close (PC/SZ-01: 29.8 MPa; PC/SZ-02: 29.1 MPa; PC/SZ-03: 24.9 MPa; PC/SZ-04: 26.8 MPa; PC/SZ-05: 24.2 MPa and PC/SZ-06: 27.4 MPa).

After 28 days of curing, the mechanical strength value of the reference specimen was reached. However, some mixed specimens significantly approached (PC/SZ-06: 50.1 MPa and PC/SZ-04: 48.9 MPa) or even surpassed it (PC/SZ-01: 51.4 MPa and PC/SZ-02: 51.3 MPa).

After 90 days of curing, the mechanical strength of the mixed specimens increased significantly (PC/SZ-01: 72.2 MPa; PC/SZ-02: 71.5 MPa; PC/SZ-04: 70.1 MPa; PC/SZ-06: 69.7 MPa), surpassing the value of the reference mortar (PCSR: 68.4 MPa). The values calculated for the remaining samples (PC/SZ-03: 65.8 MPa; PC/SZ-05: 51.3 MPa) also showed an exponential increase in compressive strength.

The analysis of the mechanical behavior of the tested specimens indicated full agreement with the pozzolanicity test results given in Section 3.6. It seems to be demonstrated that the pozzolanic reactivity of the natural zeolite-rich tuff studied has a significant influence on the process of gaining mechanical strength. This fact is confirmed by several authors in their studies on mortars and concretes made with zeolites of different species [55,56,57,58].

## 4. Conclusions

The following conclusions have been drawn from the study of the zeolite samples found in the study area:

1. The results presented prove that the zeolite found in the study area is composed mostly of highly crystalline mordenite and subordinately of smectite (montmorillonite), illite, halloysite, quartz, plagioclase, and muscovite.
2. The samples analyzed have high contents of SiO_2_ and Al_2_O_3_, while the contents of alkaline compounds (Na_2_O and K_2_O) are significantly higher than those of alkaline–earth compounds (CaO and MgO).
3. The thermal behavior of the samples indicates that the mordenite is stable up to approximately 750 °C, after which it tends to collapse and restructure.
4. All the samples have shown a marked pozzolanic behavior both at 8 and 15 days, so their status as high-quality pozzolans is established.
5. It is concluded that more silica is available in cement to natural zeolite-rich tuff mortars than in those made exclusively with cement. This availability of SiO_2_ could lead to an increase in mechanical strengths in the long term.
6. The SiO_2_/(CaO + MgO) ratio is high, which favors the pozzolanic reaction since the silica phase will tend to react with the alkaline phase.
7. Natural zeolite-rich tuff influences the balance of the SO_3_ content in mortar samples, causing this compound to always remain in solution and prevent ettringite from forming in abnormal quantities.
8. The presence of zeolite in mixed mortar mixes (PC/ZS) favors the increase of mechanical strength from 7 to 90 days. During this period, the strength values equaled or even exceeded the reference mortar. In this study, Portland cement was replaced by 25% natural zeolite-rich tuff. However, it is possible that with formulations of PC/ZS: 70–30%, relevant results can also be obtained.
9. According to the points argued above, it is established that the natural zeolite-rich tuff studied is qualitatively suitable for the improvement of cements, mortars, and concretes.
10. Finally, the investigation of this new finding corroborates that this natural zeolite-rich tuff has similar mineral, chemical, and technical properties that are even qualitatively better than the one found in the main deposit (San José-Los Escullos). This could be advantageous when considering the expansion of geological and mining reserves from the perspective of local industry interests.

## Figures and Tables

**Table 1:** Chemical composition of Portland cement as determined by XRF.

% Oxides Weight													
Materials	SiO_2_	CaO	Fe_2_O_3_	Al_2_O_3_	Na_2_O	SO_3_	MgO	K_2_O	TiO_2_	P_2_O_5_	MnO	PPC	Total
PC ^1^	17.45	64.04	3.35	5.59	0.091	4	0.641	1.37	0.326	0.072	0.094	2.43	99.454

^1^ Portland cement.

**Table 2:** Granulometric distribution of the normalized sand (NS) used in this research.

Square Mesh Dimensions (mm)	2.0	1.60	1.00	0.5	0.16	0.08
Residue Retained on Sieves (%)	0.00	7 ± 5	33 ± 5	67 ± 5	87 ± 5	99 ± 1

**Table 3:** Formulation of Portland cement (PC:75%)/natural zeolite-rich tuff (SZ:25%) mix ratios in mortar specimens.

Sample	Mortar Components	Formulation (g)	Test Age (Days)
PC/SZ-01 ^1^	Portland cement Natural zeolite-rich tuff Normalized sand Distilled water	Portland cement: 375 g Natural zeolite-rich tuff: 125 g Distilled water: 225 g Normalized sand: 1350 g	7/28/90
PC/SZ-02			
PC/SZ-03			
PC/SZ-04			
PC/SZ-05			
PC/SZ-06			
PCSR ^2^	Portland cement Normalized sand Distilled water	Portland cement: 450 g Distilled water: 225 g Normalized sand: 1350 g	7/28/90

^1^ PC/SZ-01 to 06: mortar specimens prepared with Portland cement and natural zeolite-rich tuff; ^2^ PCSR: mortar specimens manufactured with Portland cement only as a reference element.

**Table 4:** Chemical composition of XRF samples.

Samples	% Oxides Weight												
	S_i_O_2_	Al_2_O_3_	CaO	Na_2_O	K_2_O	MgO	Fe_2_O_3_	TiO_2_	MnO	SO_3_	LOI	Si/Al	Si/ (Al + Fe)
SZ-01	65.65	15.63	1.02	2.61	4.33	2.04	1.77	0.12	0.091	0.14	11.5	4.2	3.8
SZ-02	64.48	15.22	1.47	3.13	3.61	2.01	1.81	0.11	0.115	0.08	10.7	5.2	3.8
SZ-03	64.91	16.43	1.30	2.81	3.14	2.24	1.79	0.10	0.112	0.10	10.9	4.0	3.6
SZ-04	65.14	15.13	1.08	2.52	3.31	2.19	1.75	0.13	0.083	0.06	11.9	4.3	3.9
SZ-05	64.47	16.21	1.35	3.03	3.18	2.11	1.80	0.10	0.072	0.11	11.1	4.0	3.6
SZ-06	64.93	16.30	1.31	2.55	3.22	2.14	1.73	0.14	0.051	0.10	11.3	4.0	3.6

**Table 5:** Qualitative-technological chemical composition of the samples.

Compounds (%)	Samples					
	SZ-01	SZ-02	SZ-03	SZ-04	SZ-05	SZ-06
Total SiO_2_	69.25	68.80	68.51	68.74	68.07	68.53
Reactive SiO_2_	68.22	67.77	67.48	67.71	67.04	67.50
Total CaO	1.22	1.31	1.29	1.12	1.33	1.28
Reactive CaO	0.62	0.71	0.69	0.52	0.73	0.68
Al_2_O_3_	15.33	14.92	16.13	14.53	15.61	15.70
MgO	1.54	1.51	1.74	1.69	1.61	1.64
Fe_2_O_3_	1.36	1.40	1.38	1.34	1.39	1.32
SO_3_	0.09	0.11	0.08	0.10	0.13	0.09
I.R. ^1^	1.03	1.13	2.01	1.90	1.78	1.83
SiO_2_/(CaO + MgO)	25.0	24.4	22.6	24.5	23.1	23.5

^1^ I.R.: insoluble residue.

**Table 6:** Chemical composition of specimens made only with Portland cement at different curing ages.

Sample		% Oxides Weight									
	S_i_O_2_	Al_2_O_3_	CaO	MgO	K_2_O	Na_2_O	SO_3_	Fe_2_O_3_	TiO_2_	P_2_O_5_	MnO
PC ^1^	17.47	5.60	64.05	0.63	1.35	0.09	4.00	3.31	0.33	0.07	0.09
PCS-7 ^2^	47.31	3.54	34.80	0.49	0.81	0.10	1.40	4.23	0.15	0.11	0.14
PCS-28 ^3^	45.15	3.31	37.15	0.53	0.76	0.21	1.42	4.69	0.18	0.10	0.14
PCS-90 ^4^	38.27	3.11	42.12	0.58	0.55	0.23	1.49	5.07	0.19	0.09	0.13
	130.73	9.96	114.07	1.60	2.12	0.54	4.31	-	-	-	-

^1^ Anhydrous Portland cement sample; ^2–4^ specimens of Portland cement mortar and standardized sand set at 7, 28, and 90 days.

**Table 7:** Chemical composition of specimens made with mixed mixtures of cement (75%) and natural zeolite-rich tuff (25%) at different curing ages.

Sample	% Oxides Weight										
	S_i_O_2_	Al_2_O_3_	CaO	MgO	K_2_O	Na_2_O	SO_3_	Fe_2_O_3_	TiO_2_	P_2_O_5_	MnO
SZ-01 ^1^	65.65	15.63	1.02	1.29	4.33	2.61	0.14	1.77	0.12	0.03	0.09
PC/SZ-01-7 ^2^	48.63	5.24	31.14	0.53	1.17	0.17	1.25	3.93	0.18	0.11	0.11
PC/SZ-01-28 ^3^	47.30	4.89	32.51	0.55	1.23	0.16	1.21	4.17	0.19	0.10	0.13
PC/SZ-01-90 ^4^	42.71	4.59	37.17	0.62	1.21	0.14	1.10	4.71	0.23	0.09	0.15
	138.64	14.72	100.82	1.70	3.61	0.47	3.56	-	-	-	-

^1^ Sample of natural zeolite-rich tuff (SZ-01) in its natural state; ^2–4^ specimens of mixed mortars composed of cement (75%) and natural zeolite-rich tuff (25%) and set at 7, 28, and 90 days.

## Data Availability

The data are contained within the article.
